# Supplementary material for: Comparing the reliability of different ICA algorithms for fMRI analysis
Source: PLoS One. 2022 Jun 27;17(6):e0270556. doi: 10.1371/journal.pone.0270556 (PMC9236259; doi:10.1371/journal.pone.0270556)
Supplement: Attachment — Submitted filename: Response to Reviewers.docx [file pone.0270556.s003.docx]

Reviewer #1: This is a nice revision. Thank you for the work on the term ICASSO origin. This revision is coupled with a very responsive letter, but not all comments in the letter appear in the revised manuscript as I read it. Since readers may have the same concerns as reviewers it is crucial to include these in the manuscript, unless the full review history is also going to be published.

Response：Thank you very much for the comments and suggestions. The explanations in the former letter have been added in the manuscript if the contents were not included in the former version.

In particular, the issue I could not find in the materials and methods:

That the component correlation method used (in section "Comparing the reliability among four ICA algorithms with SCC") did not in the present data produce any double use correlations must be stated, and that this was observed to be true, although this is not guaranteed by the method. I still wasn't clear if the authors were relying here on other code or directly checked this in their work, and this should be clearly stated.

Response：In section "Comparing the reliability among four non-deterministic ICA algorithms with SCC” (and other sections where SCCs were calculated), the SCC was calculated by using MATLAB function *corrcoef*. We have added this information in the manuscript.

In this section, the SCC values are used in two steps.

Step 1 is a simple picking-the maximum-value process, introduced in the second paragraph of this section. This is not a statistical comparison but just picks the highest SCC value. As a result, we get a list of SCC values for each pair of groups.

Step 2 is a statistical comparison between different lists of SCCs by using the Kruskal–Wallis test, introduced in the last paragraph of this section.

Thus, this is not a double use of correlations. Step 1 provides values for statistical analysis in Step 2.

In the new version, we have added this information and highlighted supplemented words. We hope the contents will be more clearly expressed.

Line 331 - probably, term 'well-performing' is better than 'well-performed' here

Response：The word 'well-performed' has been changed to 'well-performing' based on your suggestion.
